# Non-invasive measurement of leaf water content and pressure–volume curves using terahertz radiation

**DOI:** 10.1038/s41598-020-78154-z

**Published:** 2020-12-03

**Authors:** Ran Li, Yaojie Lu, Jennifer M. R. Peters, Brendan Choat, Andrew J. Lee

**Affiliations:** 1 MQ Photonics Research Centre, Department of Physics and Astronomy, Macquarie University, North Ryde, NSW 2109 Australia; 2 Hawkesbury Institute for the Environment, University of Western Sydney, Richmond, NSW 2753 Australia; 3 Climate Change Science Institute & Environmental Science Division, Oak Ridge National Laboratory, Oak Ridge, TN 37831 USA

**Keywords:** Plant ecology, Plant physiology, Plant stress responses, Polaritons, Terahertz optics

## Abstract

In this paper we describe a non-invasive method of measuring leaf water content using THz radiation and combine this with psychrometry for determination of leaf pressure–volume relationships. In contrast to prior investigations using THz radiation to measure plant water status, the reported method exploits the differential absorption characteristic of THz radiation at multiple frequencies within plant leaves to determine absolute water content in real-time. By combining the THz system with a psychrometer, pressure–volume curves were generated in a completely automated fashion for the determination of leaf tissue water relations parameters including water potential at turgor loss, osmotic potential at full turgor and the relative water content at the turgor loss point. This novel methodology provides for repeated, non-destructive measurement of leaf water content and greatly increased efficiency in generation of leaf PV curves by reducing user handling time.

## Introduction

The determination of plant and leaf water relations characteristics is of critical importance to physiological ecology and agricultural sciences ^1–4^. Within the context of water management and assessment of plant drought tolerance, the osmotic potential at full turgor ($\uppi _{{\text{o}}}$) and water potential at turgor loss ($\Psi_{{{\text{tlp}}}}$) ^5–7^, are the most commonly examined characteristics. These characteristics are typically determined through the generation of pressure–volume (PV) curves, wherein the bulk leaf water potential of the leaf ($\Psi_{{\text{L}}}$) is plotted as a function of the leaf relative water content (RWC).

The relative water content of a leaf has traditionally been measured using a high-sensitivity balance in a gravimetric weighing process, wherein the RWC is given as a ratio of the current leaf water content over leaf water content when fully saturated ^8^. This measurement requires the removal of a leaf (or stem) sample from the plant, hydration of the sample and complete drying of the sample. This process in itself is destructive and time-consuming. Similarly, the determination of water potential is a destructive method, wherein, the Scholander pressure chamber ^7,9^, has become the device of choice ^10–12^.

Measurement of water potential using the Scholander pressure chamber is a labour-intensive process where a skilled operator is required to repeatedly pressurise and de-pressurise a chamber in which the leaf sample is housed, and the operator must carefully determine the pressure at which water emerges from the leaf petiole. This method is however, subject to a number of potentially significant sources of error ^7,13,14^. Alternative methods of estimating plant water potential include psychrometry (or hygrometry) ^13,15–17^, pressure probes ^18^, measuring leaf thickness ^19,20^, and osmometry ^21^. Of these methods, psychrometry offers one of the most flexible methods of measuring water potential, being easily interfaced with plant trunks, stems, and leaves ^13,22^. There have been numerous studies demonstrating excellent correlation between water potential measurements taken using psychrometers, in comparison to that taken using the Scholander pressure chamber ^12,17,23^. Some deviations from 1:1 correlation between the two measurement methods have been observed, the reasons for which, include variation in leaf morphology, differences in water potential through the leaf, and epidermal conductance ^16^.

Given the maturity of psychrometer technology, surprisingly it has not been adopted more broadly as a method of measuring water potential for PV curves. One limitation may be measurement difficulty when used on species with high levels of resin or mucilage. There may also be perceived complexity when used for the generation of PV curves, wherein, the psychrometer may need to be detached and reattached for concurrent measurements of leaf mass ^12^. This may be simply overcome through the use of a precision balance to constantly monitor the complete system weight of the psychrometer attached to the leaf sample. Nevertheless, collecting a standard PV curve still requires the detachment of a leaf sample from the plant in order to conduct these measurements. A non-destructive assay of leaf water content would allow for the generation of leaf PV curves in situ.

The application of THz and sub-THz radiation to the determination of plant water status has been highlighted by a number of groups ^24–31^, each of whom have made use of the strong differential absorption of radiation at these frequencies by water, in contrast to absorption by plant material. A majority of these studies have made use of radiation in the 150–300 GHz range ^25,27,28^, as this is the frequency range over which their sources produced the most power (with average powers in the nano-Watt range) ^25^. Many THz sources including THz time domain spectrometers (THz-TDS) and THz quantum cascade lasers are commercially available, and are becoming ever-more affordable as advances in laser and semiconductor technologies advance. In many respects, THz application utilising these THz sources are now primed to transition from the lab and into the “real-world”. One group has made use of THz radiation from a THz-quantum cascade laser (QCL), at a frequency of 2.5 THz, and have compared their measurements with gravimetrically-derived relative water content ^24^. Each of these methods have simply examined THz absorption at a single frequency (or the collective power across a broad frequency band, in the case of THz-time-domain based systems), for the determination of water content.

While these investigations applying THz radiation to the measurement of water content in plants have proven insightful, they do not fully leverage the capabilities of emergent THz sources. Specifically, prior studies have not made use of differential absorption of THz radiation by components within the interrogated leaves. Absorption of THz radiation by water varies with THz frequency ^32^ and by carefully examining this differential absorption characteristic, a more powerful, and instantaneous methods for determining plant water content can be developed. However, this does require a THz radiation source with high spectral-brightness, and this is a characteristic of intracavity THz sources based on stimulated polariton scattering ^33–36^, which have average output power in the 0.1 mW range, with broad frequency tunability across the range 1–6 THz ^34–36^.

In this paper, we demonstrate the effectiveness of a multi-frequency THz interrogation approach to determine water layer thickness within leaf samples, and we propose the use of THz radiation in combination with psychrometer measurements to dynamically produce PV curves of leaf samples in situ (on plant), wherein the water potential of plant leaves, in relation to their relative water content can be monitored. This method exploits the non-contact and non-destructive characteristics of measuring leaf water content using THz radiation. To the best of our knowledge, the in situ determination of PV relations is a process/methodology which has never been previously proposed, and has the potential to offer new insights into plant physiology. This methodology is presented in the following structure: a) demonstration of the use of frequency-tunable THz radiation for the measurement of leaf water layer thickness using two different approaches (the first requiring complete drying of a representative leaf, and the other exploiting the multi-frequency capability of our THz source); b) comparing THz-derived relative leaf water content with that measured gravimetrically; c) measurement of PV curves in a sample plant species (*Vitis vinifera*), and determination of osmotic potential at full turgor and turgor loss within these samples.

## Materials and methods

### THz source and optical setup

**Figure 1:**
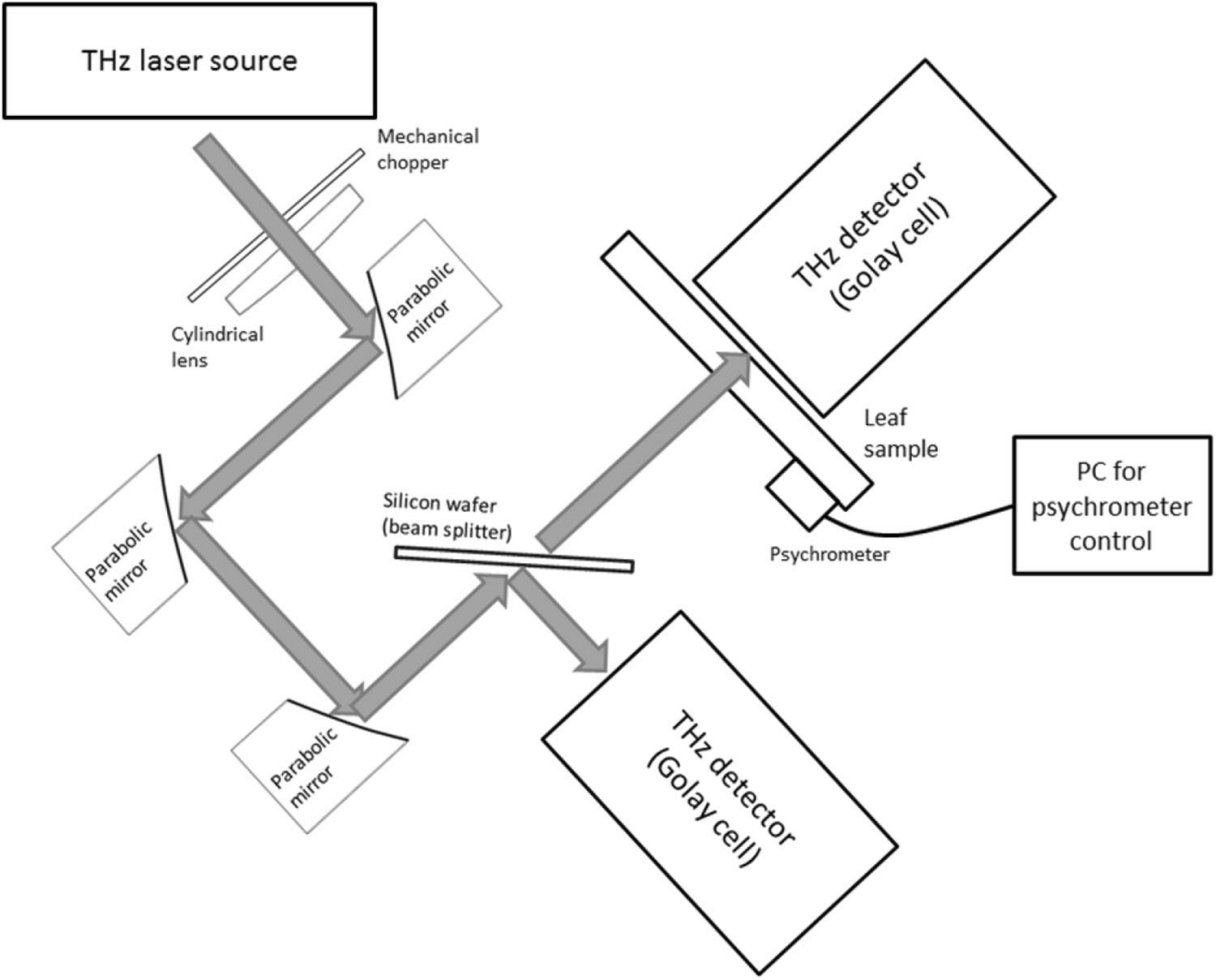
Experimental setup for interrogation of leaf samples using THz radiation. The THz laser beam path is depicted by the grey arrows.

The THz source utilised in this work was an intracavity stimulated polariton scattering (SPS) laser based on an Nd:YVO_4_ laser crystal and an 5% at. MgO:LiNbO_3_ SPS-active crystal. The laser made use of the fundamental emission line at 1342 nm in the Nd:YVO_4_ crystal, and produced an average THz output power of 20 µW at 5 kHz pulse repetition frequency. The THz emission linewidth was ~ 0.05 THz. The exact specifications and setup of this laser source are detailed in ^37^. The THz radiation was collimated and then focussed using a combination of one cylindrical lens (Tsurupica, RR-CX-100-SPS-CL); to collimate the THz beam in the vertical direction, and three gold coated parabolic mirrors (Thorlabs, MPD249-M01). The experimental setup is shown in Fig. 1. The THz radiation was chopped using a mechanical chopper (Thorlabs, MC2000B) at a frequency of 10 Hz (50% duty cycle). An optically polished, high-resistivity silicon wafer (50 mm diameter, 500 µm thickness) was used as a beam splitter (50:50 split) to enable simultaneous monitoring of a reference THz beam, in addition to the THz probe beam, as a means of compensating for any fluctuations in THz output. The THz radiation was focussed to a spot diameter of 500 µm ± 50 µm, on the surface of the leaf sample. The two split THz fields were detected using two calibrated Golay cells (Tydex, GC-1T). The THz signal was collected from the Golay cells using a digital-analogue-converter (National Instruments, USB 6211) interfaced with a PC, using custom LabVIEW code.

Frequency-tuning of the THz system was automated and controlled using a mechanical rotation stage (Thorlabs, KPRMTE), which controlled the angle of the Stokes field resonator relative to the fundamental field resonator, enabling frequency tuning across the range 1.1–1.8 THz in 0.005 THz increments ^34^.

In this study, the THz laser source was tuned to a frequency of 1.3 THz, and an average power of 20 μW was incident on the leaf samples. The THz beam was focused to a spot of diameter 500 μm, at a position mid-way along the length of the leaves, and approximately 2 mm from the mid-rib of the leaf (in the leaf lamina).

### Plant material

The leaf samples examined in this work were of 3 different plant species: *Sloanea australis* (maidens blush), *Eucalyptus haemastoma* (scribbly gum), and *Vitis vinifera* (common grapevine). All leaf samples (7 of each species) were collected from healthy, non-droughted plants and directly bagged (in polyethylene zip-lock bags) in the morning between 11 am and 12 pm. The leaves were mature, fully expanded, and healthy with a uniform green appearance with no signs of yellowing.

Leaf samples of *Sloanea australis* were taken from a well-established tree (approximately 15 years old) on the Macquarie University campus. The tree had been growing under natural conditions and appeared healthy, with a height of approximately 3 m. The leaf samples were collected from the lowest branch of the tree at a height of approximately 1 m. The leaves were approximately 15 cm in length and 5 cm in width.

Leaf samples of *Eucalyptus haemastoma* were collected from a well-established tree (approximately 10 years old) on the Macquarie University campus. The tree had been growing under natural conditions and appeared healthy, with a height of approximately 5 m. The leaf samples were collected from the lowest branch of the tree at a height of approximately 2 m. The leaves were approximately 9 cm in length and 4 cm in width.

Leaf samples of *Vitis vinifera* were taken from a well-established grapevine (approximately 20 years old), which had grown under natural conditions close to the Sydney harbor side (Seaforth). The leaf samples were collected from parts of the vine at a height of approximately 1.5 m. The leaves were approximately 13 cm in length and 12.5 cm in width.

### Measurement of water layer thickness using THz radiation

The application of THz radiation in the determination of water content relies on differential absorption of the incident THz radiation by the water within the leaf samples and that of the leaf material. The effective medium model proposed by ^38^, considers the total leaf structure as a composite of water, leaf matter and air. In this work, a simplified model wherein the leaf structure is assumed to comprise only water and leaf material, has been used.

In this work, the THz radiation incident on the leaf sample, and that transmitted through the leaf sample is measured, in order to calculate a leaf transmission ratio (T, the ratio of the transmitted THz power to incident THz power). Based on Beer-Lambert absorption ^39^, this transmission ratio decreases exponentially as a function of the thickness of the water layer and that of the leaf material layer, and can be expressed as

1 $$ T = e^{{ - \alpha_{w} d_{w} }} \times e^{{ - \alpha_{l} d_{l} }} , $$

where *T* is the THz transmission ratio (THz power transmitted through the leaf divided by the THz power incident on the leaf), *α*_*w*_ is the absorption coefficient of water at the probed THz frequency, *d*_*w*_ is the water layer thickness at the leaf location probed by the THz beam, *α*_*l*_ is the leaf material absorption coefficient at the probed THz frequency, and *d*_*l*_ is the leaf material thickness.

This equation provides a means of using the total THz transmittance through the leaf to determine both the thickness of the water layer in the leaf, and the thickness of the leaf material, along with the absorption characteristics of each. The THz absorption characteristic of water is well known ^32^, and it has been shown that this is largely invariant within plant leaves, where internal liquid may contain proteins, peptides, amino acids, hormones and carboxylates ^40^. By plotting the natural logarithm of the THz transmission ratio ($ln\left( T \right)$) with leaf weight loss (determined gravimetrically), we demonstrate that the quantity $\alpha_{l} d_{l}$, remains constant as the leaf dries out. Given that the leaf material absorption coefficient (*α*_*l*_) is constant at a fixed THz frequency, this demonstrates that the leaf material thickness also remains constant as the leaf water content changes. We have also shown that the quantity $ln\left( T \right)$ is linear with gravimetric weight loss, as the leaf dries out, demonstrating that the quantity $ln\left( T \right)$ may be used as a proxy for gravimetrically determined leaf weight (refer to **Invariance of** ${\varvec{\alpha}}_{{\user2{l }}}$ **and the linearity of ln(T) with gravimetric weight loss**, and Fig. 3).

### Determination of absolute water layer thickness with complete drying of the leaf sample

The absolute thickness of the water layer can be derived gravimetrically ^41^ using the following equation,

2 $$ \begin{aligned} & Effective \,water\, layer \,depth \left( {averaged \,across\, the\, entire\, leaf} \right) \\ & \quad = \frac{Water \,mass\, in \,the\, leaf}{{Density\, of \,water}}.\frac{1}{Surface \,area\, of \,the \,leaf}, \\ \end{aligned} $$

In the case where THz transmission measurements are made, and the leaf is monitored to a state where it is completely dry (for example through accelerated drying in an oven), the water layer thickness may be determined through the following equation,

3 $$ d_{W} = \frac{{Ln\left( T \right) - Ln(T_{D} )}}{{ - \alpha_{W} }}, $$

where T is the THz transmission ratio measured at a given time, T_D_ is the THz transmission ratio measured through a fully dry leaf, and *α*_*w*_ is the absorption coefficient of water at the probed THz frequency. These measurements were performed across all investigated species, *Sloanea australis*, *Eucalyptus haemastoma*, and *Vitis vinifera*.

### Direct measurement of water absorption coefficient

The absorption coefficient of water was directly measured in leaf samples of *Sloanea australis*, *Eucalyptus haemastoma*, and *Vitis vinifera* with the use of the THz source, in combination with gravimetric measurements, and water layer thickness as derived using Eq. (2). By taking two THz transmission measurements (T_1_ and T_2_) at two different times 1 and 2, we can derive an equation for the water absorption coefficient:

4 $$ T_{1} = e^{{ - \alpha_{w} d_{w1} }} \times e^{{ - \alpha_{l} d_{l} }} , $$

5 $$ T_{2} = e^{{ - \alpha_{w} d_{w2} }} \times e^{{ - \alpha_{l} d_{l} }} , $$

The simultaneous solution of these equations yields the following expression for the absorption coefficient of water at the given THz frequency:

6 $$ \alpha_{w} = \frac{{ln\left( {\frac{{T_{2} }}{{T_{1} }}} \right)}}{{\left( {d_{w1} - d_{w2} } \right)}}, $$

**Figure 2:**
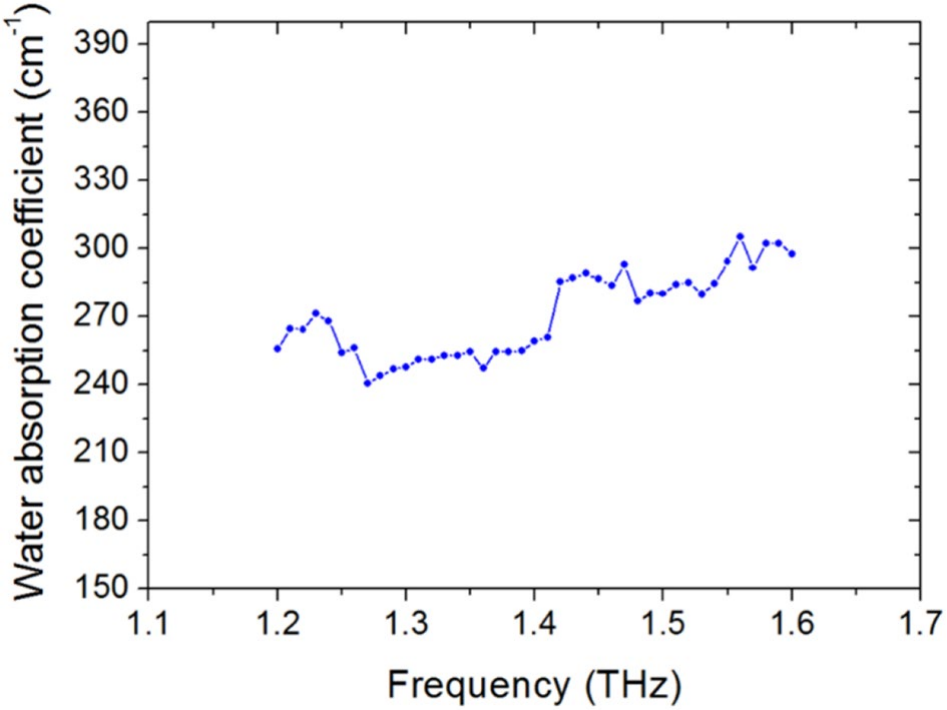
Plot of absorption coefficient of water, as determined within a sample of *Sloanea australis*, as a function of THz frequency, over the range 1.2–1.6 THz.

The values of *d*_*w1*_ and *d*_*w2*_ were determined through direct gravimetric weight measurements, immediately following the THz transmission measurement. This process was repeated for THz frequencies across the range 1.2–1.6 THz. A plot of the water absorption coefficient in this range, as determined within a leaf sample of *Sloanea australis* is shown in Fig. 2.

### Combined THz and gravimetric measurements

In this work, sequential measurement of THz transmission, and gravimetric weight measurements of leaf samples were taken. In this case, leaves were mounted in a metal framework and secured in position with magnets. This ensured that the leaves could be removed from the metal frame and weighed using high-accuracy scales (Ohaus, PA214C). The leaves were marked with a permanent marker to ensure that the same position within the leaf was interrogated using the focussed THz beam, following each weighing procedure.

Prior to taking a sequence of THz and weighing measurements, the surface area of the leaf was determined through scanning the leaf and processing of this calibrated scan using ImageJ software. This was used in combination with the weight measurements and the density of water (997 kg.m^−3^) to determine an “effective water layer” averaged across the entire leaf surface, as per Eq. (2).

### Invariance of $\alpha_{l }$ and the linearity of ln(T) with gravimetric weight loss

Following Eq. (1), in the case where a leaf is taken to a completely dehydrated state, the THz transmission simply becomes:

7 $$ T_{D} = e^{{ - \alpha_{l} d_{l} }} , $$

then following Eq. (3), we can derive an equation,

8 $$ \ln \left( T \right) - \ln \left( {T_{D} } \right) = - \alpha_{w} d_{w} , $$

or equivalently,

9 $$ - \alpha_{w} d_{w} = \ln \left( {\frac{T}{{e^{{ - \alpha_{l} d_{l} }} }}} \right) = {\text{ln}}\left( {\frac{T}{{T_{D} }}} \right) , $$

**Figure 3:**
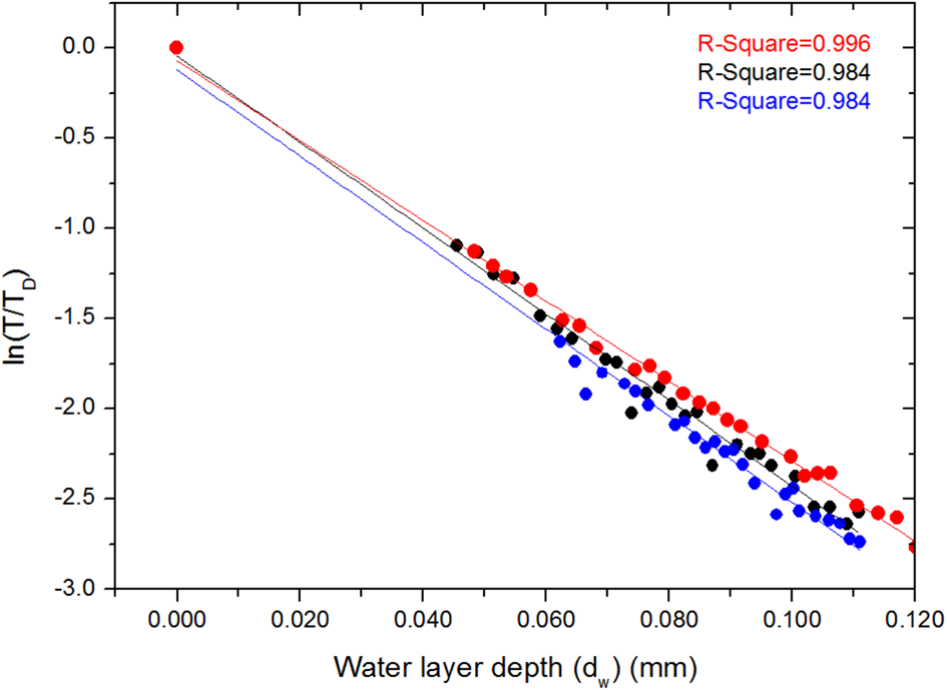
Plot of the quantities $ln\left( {\frac{T}{{T_{D} }}} \right)$ vs *d*_*w*_ for three replicates of *Solanea australis*. R-Squared values to linear fits are shown inset.

Here, the effective water layer depth (*d*_*w*_) can be derived from gravimetric measurement of the water mass in the leaf and the leaf area, as per Eq. (2). We have plotted the quantities $\ln \left( {\frac{T}{{T_{D} }}} \right)$ vs *d*_*w*_ for numerous leaf samples across a variety of plant species, and have found consistently that a linear relationship exists between these quantities across examined species, as the leaves progressively dry over time. A representative plot for 3 replicates (leaves) of *Solanea australis* is shown in Fig. 3.

The linear relationship between these two quantities shows that the value of leaf absorption (*α*_*l*_) is invariant with leaf hydration level. This also demonstrates that a linear relationship exists between the quantity ${\text{ln}}\left( T \right)$ and the gravimetrically determined leaf weight.

### Other equations used in this work

If we consider Eq. (7), along with (1) for any given point in time, the water layer thickness may be derived as follows:

10 $$ d_{w} = \frac{{\ln \left( T \right) - \ln \left( {T_{D} } \right)}}{{ - \alpha_{w} }} , $$

From this equation, it is clear that that the absorption coefficient of water (α_w_) must be known as a function of frequency, in order to derive a water layer thickness. Absorption coefficient of water across a range of THz frequencies, have been determined in this work, and is described in section “Direct measurement of water absorption coefficient”. In the case where a leaf is interrogated with two different THz frequencies, x and y, two THz transmission ratios can be calculated, with representative equations:

11 $$ T_{x} = e^{{ - \alpha_{wx} d_{w} }} \times e^{{ - \alpha_{lx} d_{l} }} , $$

12 $$ T_{y} = e^{{ - \alpha_{wy} d_{w} }} \times e^{{ - \alpha_{ly} d_{l} }} , $$

When re-arranged, these equations can be expressed as

13 $$ \frac{{\ln \left( {T_{x} } \right) + \alpha_{wx} d_{w} }}{{\ln \left( {T_{y} } \right) + \alpha_{wy} d_{w} }} = \frac{{\alpha_{lx} }}{{\alpha_{ly} }}, $$

14 $$ d_{w} = \frac{{\ln \left( {T_{x} } \right) - \frac{{\alpha_{lx} }}{{\alpha_{ly} }}ln\left( {T_{y} } \right)}}{{\frac{{\alpha_{lx} }}{{\alpha_{ly} }}\alpha_{wy} - \alpha_{wx} }}, $$

This equation is hence dependent on the ratio of leaf material absorption coefficient at frequency x and frequency y ($\frac{{\alpha_{lx} }}{{\alpha_{ly} }}$), along with the water absorption coefficients at frequencies x and y ($\alpha_{wx}$ and $\alpha_{wy}$ respectively), the values of which, may be determined using aforementioned approaches.

The determination of leaf material absorption coefficient at frequency x and frequency y, ($\frac{{\alpha_{lx} }}{{\alpha_{ly} }}$), can be derived through the simultaneous solution of THz transmission through a completely dry leaf sample at frequencies x and y:

15 $$ T_{Dx} = e^{{ - \alpha_{lx} d_{l} }} , $$

16 $$ T_{Dy} = e^{{ - \alpha_{ly} d_{l} }} , $$

Given the leaf thickness $d_{l}$ remains constant, the simultaneous solution of the two above equations, yield

17 $$ \frac{{\alpha_{lx} }}{{\alpha_{ly} }} = \frac{{{\text{ln}}\left( {T_{Dx} } \right)}}{{{\text{ln}}\left( {T_{Dy} } \right)}}, $$

### Consideration of additional attenuation factors

The methods applied in this work rely on the measurement of both the THz radiation incident on a leaf sample, and that transmitted through the sample. There is however, some reflection and scattering of the THz radiation when it is incident on the leaf sample, and as it traverses through the sample. The net effect of this reflection and scattering through the leaf can be assumed a loss factor on the incident THz radiation, which may be expressed as an attenuation factor $q$, which is the sum of fractional losses from both reflection and scattering within the leaf. A modified THz transmission ratio $T^{\prime}$ can then be defined as:

18 $$ T^{\prime} = \frac{T}{{\left( {1 - q} \right)}}, $$

where $T$ is the THz transmission ratio determined experimentally. The modified expression for water layer thickness then becomes:

19 $$ d_{wp} = \frac{{\ln \left( {pT_{x} } \right) - \frac{{\alpha_{lx} }}{{\alpha_{ly} }}ln\left( {pT_{y} } \right)}}{{\frac{{\alpha_{lx} }}{{\alpha_{ly} }}\alpha_{wy} - \alpha_{wx} }}, $$

where $$p = \frac{1}{{\left( {1 - q} \right)}}$$.

### Automation of pressure–volume curves

In these experiments, THz water content measurements were combined with psychrometer measurement of $\Psi_{{\text{L}}}$ to automate the generation of leaf pressure–volume curves. Leaf samples from *Vitis vinifera* underwent the following hydration protocol prior to measurement. Once harvested from a vine, the petiole was cut ~ 20 mm from the base of the midrib. This cut end was then secured in silicone tubing with inner diameter of 2 mm. The tubing was then filled with water and sealed using parafilm to ensure that the cut petiole was continually immersed in water. The leaves were then placed in polyethylene bags to inhibit water loss through evaporation.

The psychrometer (ICT PSY-1; ICT, Armidale NSW, AU) was interfaced with the midrib of the grapevine leaves. A thin layer of cuticle was removed to expose xylem tissue along the midrib. This region was cleaned with deionized water to remove debris and patted dry using a kimwipe. The psychrometer was then interfaced across this exposed region, oriented with the chamber positioned along the midvein and clamped in place. The chamber was sealed to the leaf using vacuum grease and the silicone tubing was removed from the leaf petiole. The approximate time between sample preparation, interfacing and first measurement was ~ 15 min. The psychrometer was set to measure the leaf water potential at 10 min intervals. Osmotic potential at full turgor ($\uppi _{{\text{o}}}$) and turgor loss point ($\Psi_{{{\text{tlp}}}}$) were calculated from plots of 1/$\Psi_{{\text{L}}}$ against RWC using standard analyses ^42^, whereby a linear regression was fitted to the linear portion of the curve and used to solve for $\Psi_{{\text{L}}}$ at RWC = 100 and $\Psi_{{\text{L}}}$ at the intersection between the exponential decay and linear portions of curve respectively.

### Mapping relative water content across the leaf surface

A map of relative water content along a line bisecting a *Vitis vinifera* leaf was taken. This experiment was undertaken so as to demonstrate the variability in relative water content across the surface of the leaf. A leaf sample was collected and hydrated following the protocol outlined in section **Leaf hydration protocol**. The leaf was then mounted using the same method outlined in the **Leaf hydration protocol** section, and then interrogated using the THz beam. Lines 16 mm long, bisecting the mid rib of the leaf, and an ancillary vein of the leaf were scanned repeatedly over the same region, with individual THz transmission data points taken at 0.5 mm intervals. The time between repeated scans at the same point in the leaf was 10 min, and the total scan time was 7 h. The leaves were then completely dehydrated over the course of 5 days in ambient lab conditions, and a second scan was taken using the same line scan parameters, in order to determine the THz transmission properties of the leaf, when fully dry. From these two data sets, the relative water content of the leaf was mapped as a function of drying time.

## Results

### Determination of absolute water layer thickness with complete drying of the leaf sample (Approach 1)

**Figure 4:**
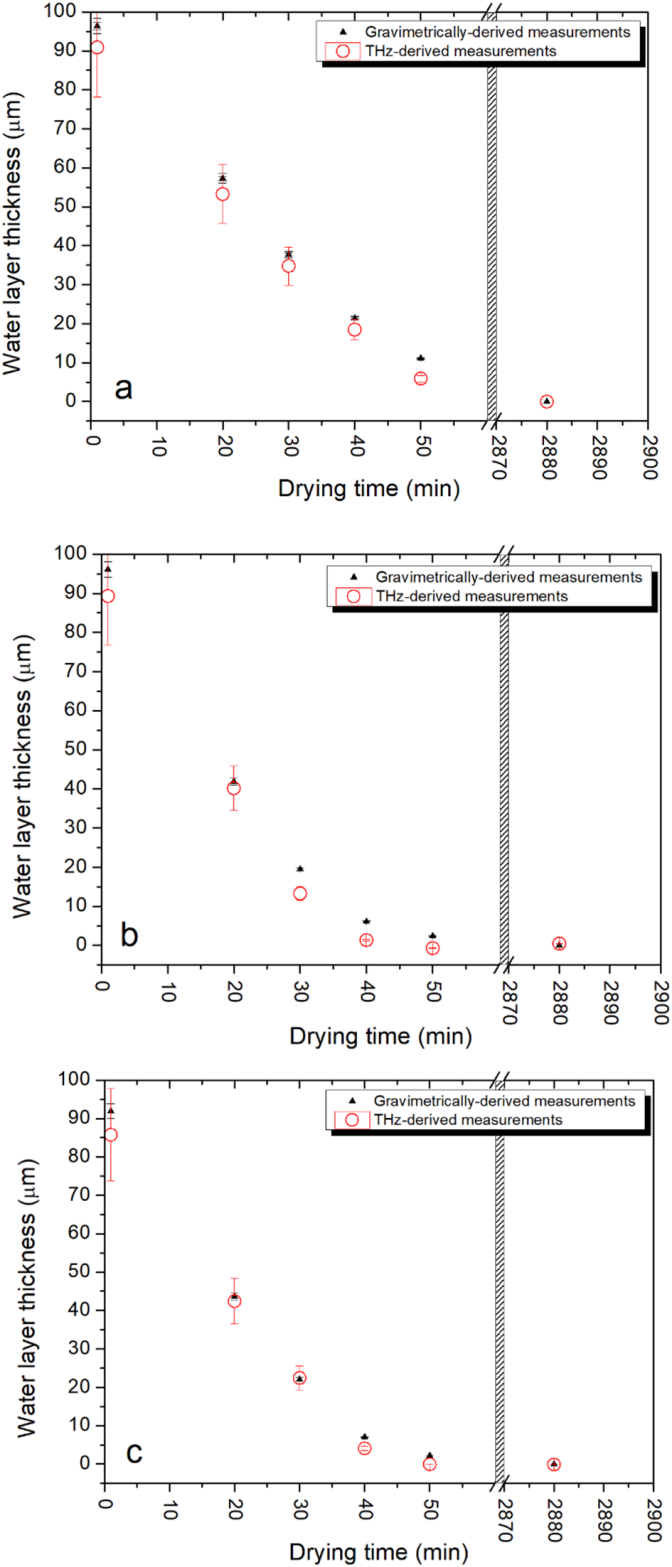
Comparison of water layer thickness as determined using the THz method (in red) with that determined using gravimetric weight measurements (in black), both as a function of drying time. Figures (**a**)–(**c**) show results from three different leaf samples (replicates) of *Sloanea australis*. Note that the samples underwent accelerated drying in an oven for a total of 50 min. An additional data point was taken when the samples were completely dried after a period of 2880 min.

The water layer thickness (*d*_*w*_) in leaves from *Sloanea australis* were measured using the gravimetric approach (and Eq. (2)), along with measurements of THz transmission ratio, and Eq. (3). Note that we directly determined the absorption coefficient of water within these leaves across the range 1.2–1.6 THz; these values compare very well with water absorption coefficients published in ^32^. Both THz and gravimetric measurements of the leaves were taken at discrete time intervals, following periods of accelerated drying in an oven (drying intervals of 10 min at 70 °C). Measurements were taken across 3 replicates and results are shown in Fig. 4.

The plots of Fig. 4 show that the results obtained using the THz transmission measurements and those taken using gravimetric measurements track well with one another. These results support previous observations that THz transmission can be used very effectively as a direct determinant of leaf water content ^28^. It should be noted that the error in the THz-derived water layer thickness (red dots) increases with water layer thickness. This is a result of the increase in associated error in transmitted THz signal, when the signal strength is low.

### Determination of absolute water layer thickness without the need for complete drying of the leaf sample (Approach 2)

The prior section demonstrated that it is possible to utilise THz radiation to effectively determine leaf water thickness, provided the leaf sample is completely dried, in order to completely differentiate the absorption characteristic of the plant material, in contrast to that of the water. However, this process is somewhat inconvenient, given the need to completely dry the leaf sample (to yield a dry leaf thickness, *d*_*l*_), and in this regard, it does not offer a significant advance over conventional gravimetric approaches.

A far better approach is to utilise the differential absorption characteristic of THz radiation in water, at different THz frequencies. By taking discrete THz transmission ratio measurements at two different frequencies, *x* and *y*, we may determine the water layer thickness (*d*_*w*_) using Eq. 14. Note that the equation has no dependence on the dry leaf material thickness $d_{l}$. This implies that no knowledge of leaf material thickness is required for determination of water layer thickness using this approach.

The expression requires the determination of the ratio of leaf absorption coefficients $\frac{{\alpha_{lx} }}{{\alpha_{ly} }}$, which is species specific, and can be determined for a completely dry leaf sample, for a given species. As detailed in the “Materials and Methods” section, it is possible to form an equation wherein the ratio of absorption coefficients $\frac{{\alpha_{lx} }}{{\alpha_{ly} }}$ can be determined simply through the ratio of THz transmission through the dry leaf at frequencies x and y, i.e.,

20 $$ \frac{{\alpha_{lx} }}{{\alpha_{ly} }} = \frac{{{\text{ln}}\left( {T_{Dx} } \right)}}{{{\text{ln}}\left( {T_{Dy} } \right)}}, $$

This equation dispenses with the requirement of measuring dry leaf material thickness and could be performed on a representative sample prior to a series of measurements, for example, across a crop of the same species. Values of water absorption coefficient (*α*_*w*_) may be taken from the literature or determined experimentally as done in this work.

**Figure 5:**
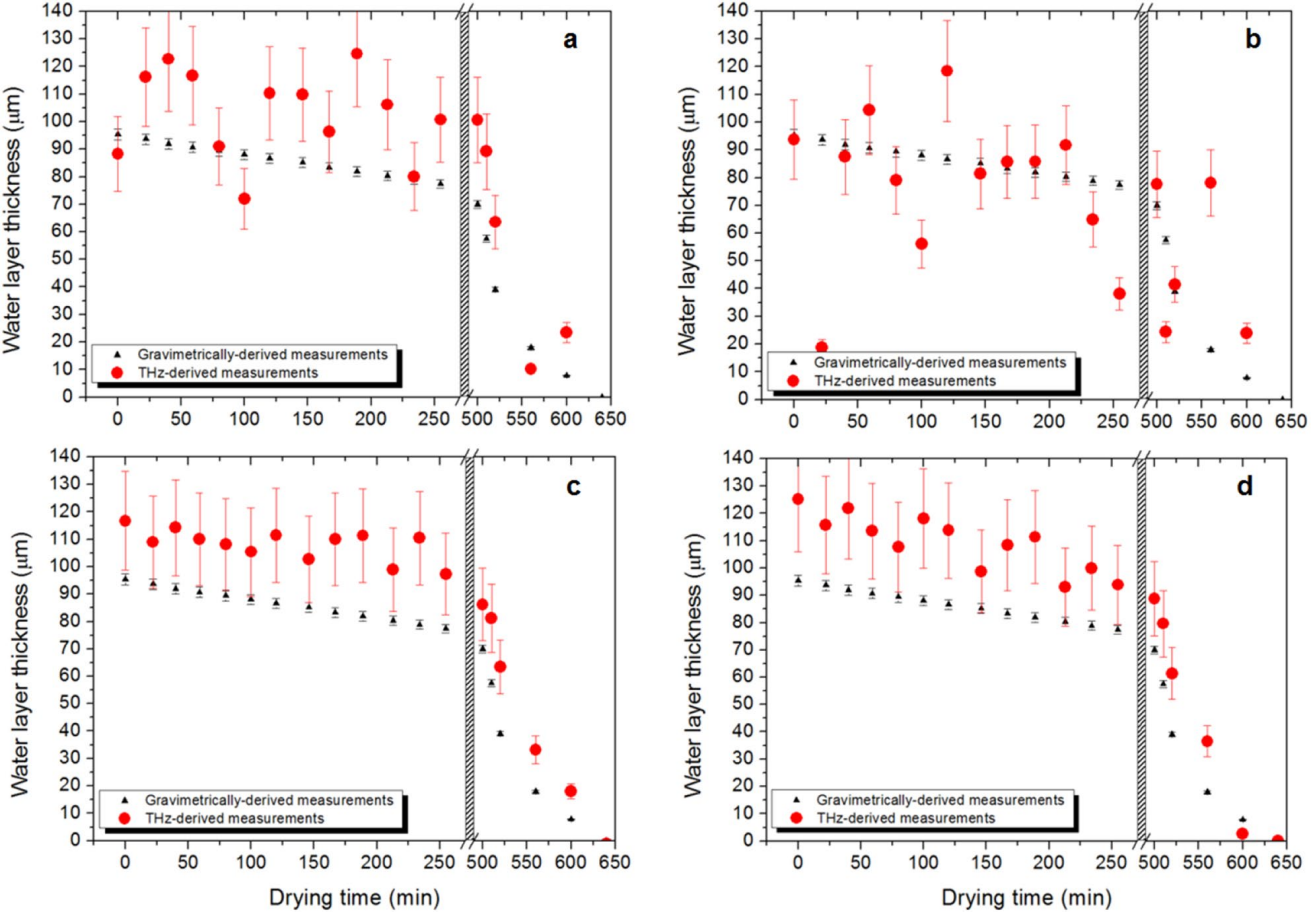
Plots of water layer thickness as determined using the THz approach (in red), and gravimetric measurements (in black), as a function of drying time in a sample of *Sloanea australis*. Note that measurements were taken up to 250 min, when drying under ambient laboratory conditions (typically 23° C, 40% relative humidity). All measurements taken after 500 min were done so with the leaf undergoing accelerated drying in an oven (70° C). The THz measurement and weight measurements were taken within 2 min of one another, ensuring minimal change in water content between the two measurement methods. Plot (**a**) shows results for 1 pair of frequencies; (**b**) 3 pairs of frequencies; (**c**) 36 pairs of frequencies; and (**d**) 465 pairs of frequencies.

Using this approach, the water layer content across 3 replicates of *Sloanea australis* was measured and compared to results obtained using gravimetric measurements. To enable these measurements, the absorption coefficient of the leaf material, as a function of THz frequency was determined using completely dry samples (see “Materials and Methods”). Also, the broad frequency-tunability of our THz laser source was utilized to perform averaging across multiple pairs of THz frequencies, the effect of which is shown in Fig. 5.

The results of Fig. 5 show that the THz-derived and weight-derived measurements of effective water layer thickness follow the same trend as the measurement number increases. Also, as the numbers of pairs of frequencies used, increases, there is a significant reduction in the scatter of the THz-derived data points. There is, however, an offset between the THz- and gravimetrically-derived values, wherein the THz-derived measurement is giving consistently higher overall water layer thickness, than the gravimetrically-derived measurements.

**Figure 6:**
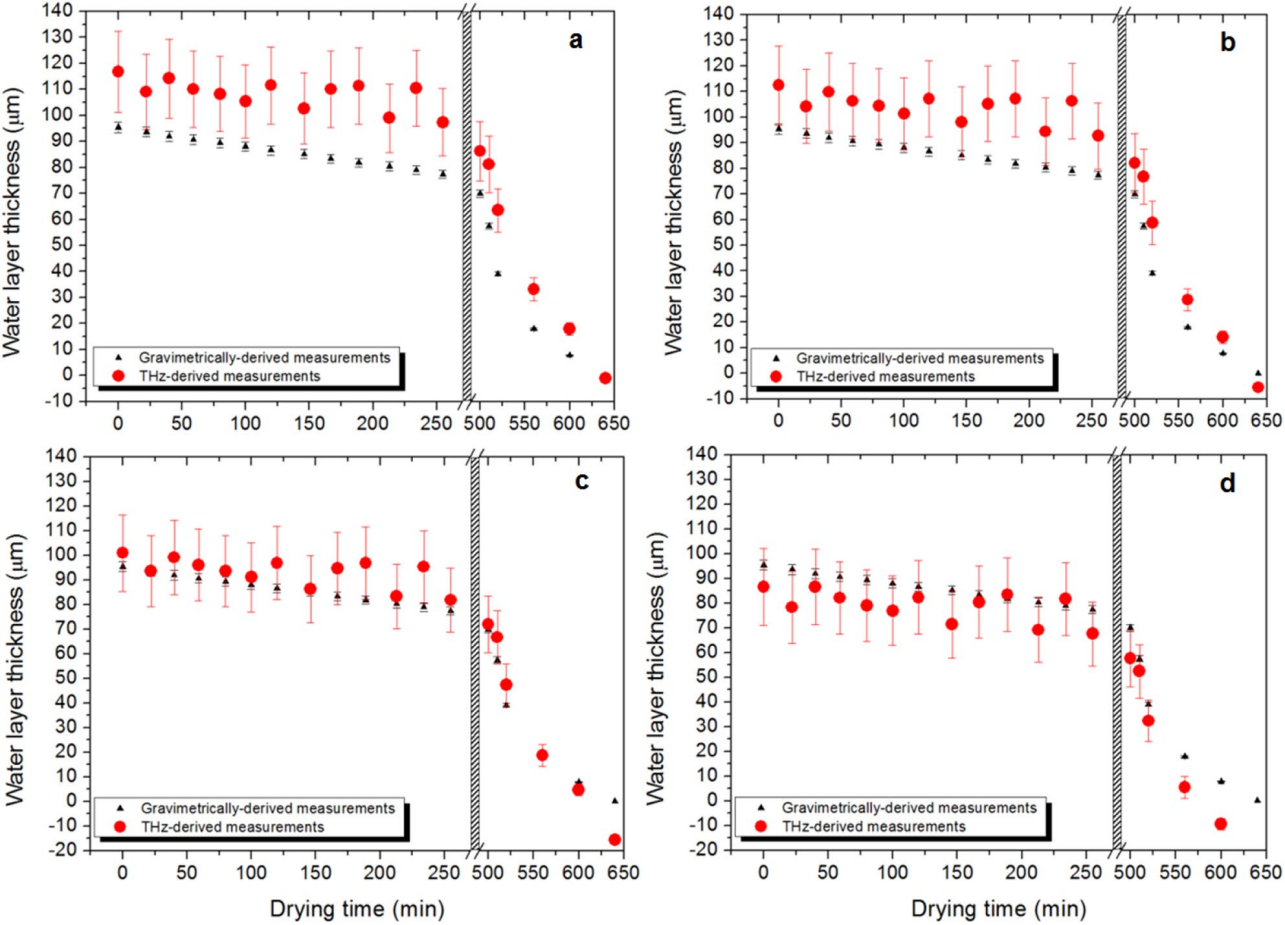
Plots of THz- and gravimetrically-derived water layer thicknesses as a function of drying time, for different values of attenuation of the incident THz laser beam in a sample of *Sloanea australis*. THz-derived results are shown in red, while gravimetrically-derived measurements are shown in black for additional attenuation factors (q), of (**a**) 0%; (**b**) 10%; (**c**) 30%; and (**d**) 50%. The drying time characteristics are as detailed in the caption of Fig. 5.

These measurements have been refined through a consideration of additional losses which occur to the THz beam, manifesting as reflection and scattering losses, when the beam is incident on, and propagates through the leaf sample. We have examined the THz reflection properties of a number of leaf samples, and the reflected THz signal within the THz frequency range of 1.3–1.6 THz varies between 10–30%, depending on species. We have adjusted our THz-derived model (refer to “Materials and Methods”) to include an additional attenuation factor ($q$) which takes into consideration reflection and scattering losses, and with this adjustment, we derive water layer thicknesses much closer to those derived using the gravimetrically-derived method as shown in Fig. 6 for samples of *Sloanea australis*.

### Pressure–volume curve analysis

Relative water content (RWC) ^43^ is typically calculated based on measurements of current leaf weight (LW_curr_), combined with dry leaf weight (LW_dry_) and saturated leaf weight (LW_sat_), the equation being,

21 $$ RWC \left( \% \right) = 100 \times \left( {\frac{{\left( {LW_{curr} - LW_{dry} } \right)}}{{\left( {LW_{sat} - LW_{dry} } \right)}}} \right), $$

Following this definition of RWC, we derive an expression for RWC, as determined using THz transmission ratios. From the derivation, it follows that the RWC, can be simply represented as:

22 $$ RWC \left( \% \right) = 100 \times \left( {\frac{{d_{w\_curr} }}{{d_{w\_sat} }}} \right), $$

where *d*_*w_curr*_ and *d*_*w_sat*_ are the current and saturated leaf water layer thicknesses, as determined using the THz-derived approach. So, if a plant has a number of representative leaves, it is possible to perform an initial study of the saturated leaf water layer thickness (wherein the water layer thickness in a representative sample is fully-hydrated), and use this as a basis to convert all current water layer thickness measurements into relative water content measurements.

**Figure 7:**
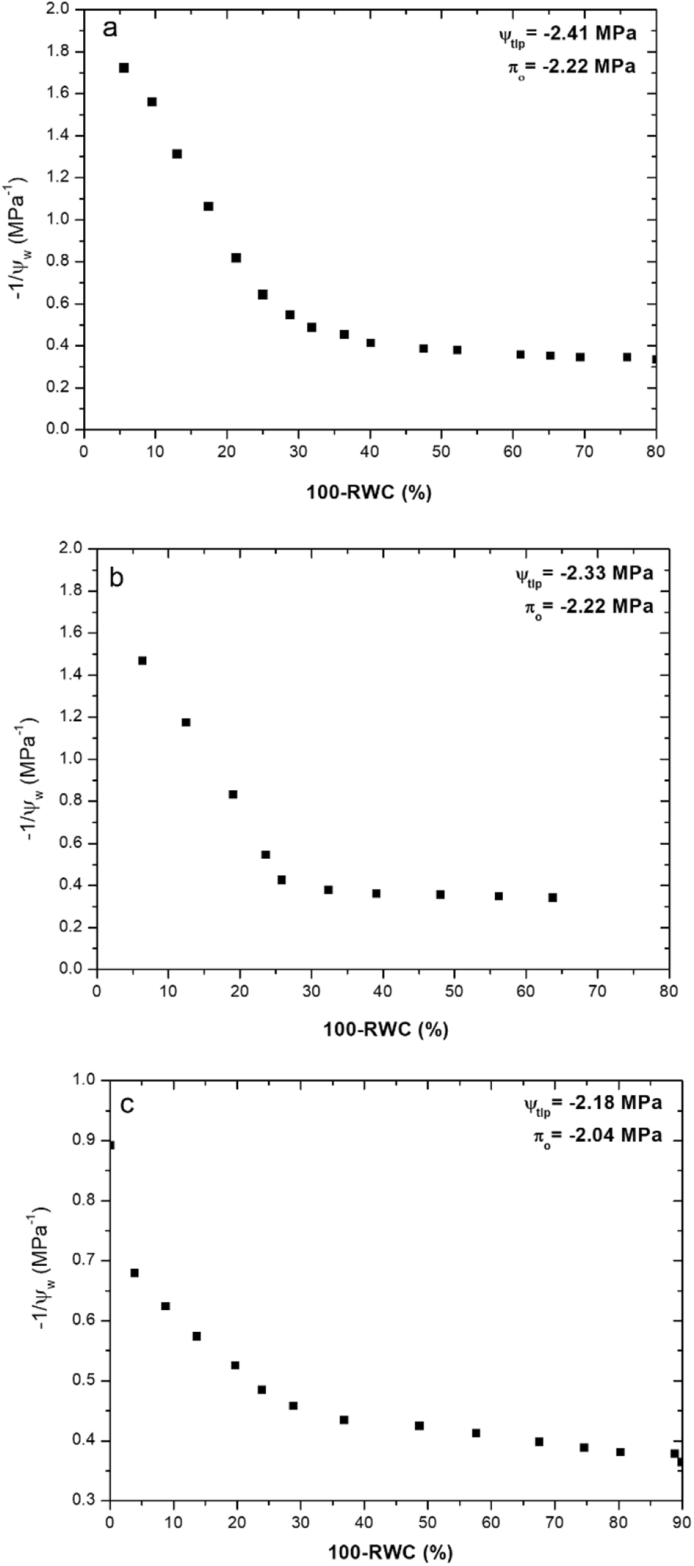
PV curves for *Vitis vinifera* leaves across three replicates (**a**), (**b**) and (**c**), determined using the THz and psychrometer approach.

The utility of RWC measurements becomes apparent when measuring additional parameters of interest in plant physiology. In this work, a stem psychrometer (ICT, PSY-1) was used in conjunction with THz measurements of RWC, to generate pressure–volume curves of leaf samples from *Vitis vinifera*, to determine $\uppi _{{\text{o}}}$ and $\Psi_{{{\text{tlp}}}}$. Representative PV curves using this technique along with values of $\uppi _{{\text{o}}}$ and $\Psi_{{{\text{tlp}}}}$ determined from these curves are shown in Fig. 7.

The measurements of $\Psi_{{{\text{tlp}}}}$, derived using the method (as highlighted in Figs. 7 (a)-(c)), are in the range of values which have been reported in the literature for *Vitis vinifera* (-1.72 to -2.42 MPa) ^44,45^.

## Discussion

The results presented here demonstrate that THz-transmission measurements taken across a range of different THz frequencies, can be very effectively used to determine leaf water layer thickness, without the need for complete drying of the leaf, provided that the THz transmission properties of a completely dry, representative leaf are taken prior. The power and novelty of this THz-derived method lies in the fact that the absolute water thickness levels may be determined in situ, without needing to remove leaf samples from the plant. This is highly relevant in circumstances where the hydration level of plants may need to be determined for high-value/fragile species ^46^, or in individual plants that have a limited availability of leaf samples. Furthermore, absolute water level measurements may be converted to relative water content, when combined with measurements of water layer thickness on representative, fully hydrated/saturated leaf samples.

Additionally, we demonstrated the potential for combining THz measurements with psychrometry to automate the generation of leaf pressure–volume curves. Using this approach, we have determined values of turgor loss point in *Vitis Vinifera*, which compare well with those reported previously for *V. vinifera* in the literature. Automation of data acquisition for both RWC and $\Psi_{{\text{L}}}$ allows for a significant increase in the number of data points that can be recorded in each PV curve. This increase in temporal resolution results from the automated “set and forget” nature of the THz and psychrometer measurement process, wherein data can be taken repeatedly at user-defined times without the need to disturb the sample. This is in stark contrast to traditional, user-intensive methods of generating PV curves which make use of a Scholander pressure chamber to repeatedly measure the leaf water potential and scales to determine the leaf weight loss. It also reduces the potential for experimental artefacts associated with damage to leaf samples from repeated pressurisation-depressurisation cycles and compression of the petiole by the pressure chamber gasket.

**Figure 8:**
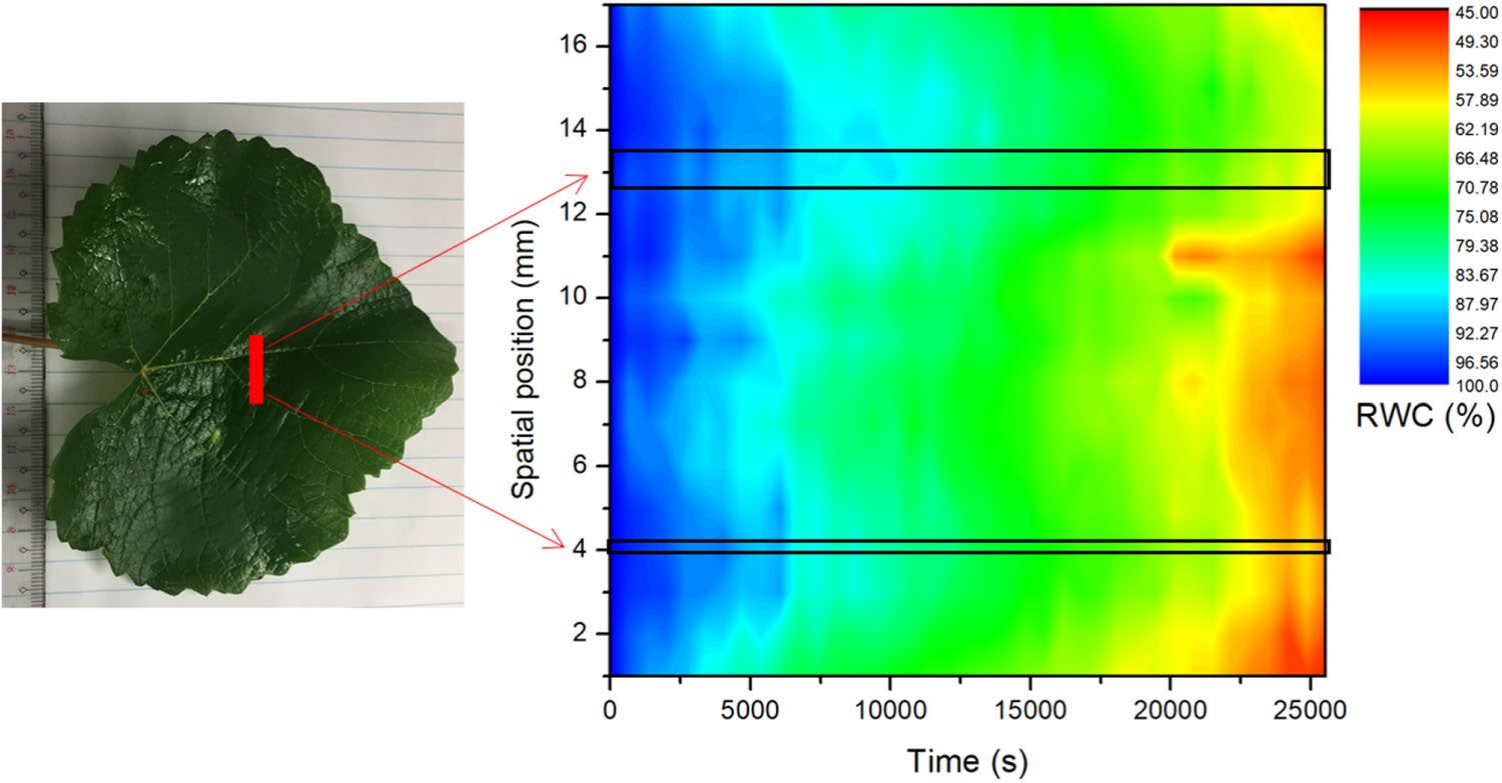
Spatial variation in RWC as a function of drying time, taken as repeated scans in a line across a leaf sample of *Vitis vinifera*. The photograph on the left (shown inset) indicates the region (in red) across which the THz transmission measurements were made in a repeated manner, as the leaf dried in ambient laboratory conditions. In the plot, the midrib occupies the region of the scan at ~ 13 mm, and the region of a vein is at ~ 4 mm.

Using THz radiation, we also demonstrated that the rate at which water content, and hence RWC changes, varies spatially throughout the leaf. This characteristic can be visualised using THz radiation, wherein, the THz transmission through the leaf changes as the leaf dries out, in accordance with decreasing water content. Shown in Fig. 8 is a false-colour intensity plot of RWC, as determined using the THz transmission approach, taken in a transect across a leaf sample (*Vitis vinifera*), as a function of drying time (in ambient laboratory conditions). An image of the leaf is shown with the position of the vertical line scan in red, which begins in the lamina, traverses the midrib, and into the lamina on the opposite side of the midrib and through another vein. This scanning process was repeated as the leaf dried, allowing for high resolution spatial and temporal analysis of changes in RWC.

The results clearly show that the RWC is not uniform across the leaf, and the RWC changes at different rates across the leaf, as it dries out. Specifically, measurements of RWC were consistently higher when taken closer to the midrib or a vein, in comparison to that of the lamina, at any given drying time. Given that the area measured by THz was located in the lamina (THz spot diameter ~ 500 μm), it follows that a lower value of RWC would be expected when comparing to bulk leaf RWC measured gravimetrically. Higher water content in the midrib and petiole at a given timepoint would likely bias this measurement towards higher values of RWC than are present in mesophyll and epidermal cells. Some support for this is provided from cell pressure probe data, which suggest that individual mesophyll and epidermal cells lose turgor at lower RWCs (70–75% of initial cell volume) than are typically calculated using PV curves (85–90% RWC) ^47,48^. Further work using THz radiation has the potential to improve our understanding of how leaf cell water status and water content varies spatially and temporally during dehydration.

## Conclusions

In this work we have demonstrated the application of tunable THz radiation to the determination of leaf RWC and in the generation of leaf PV curves. The results demonstrate the enormous potential of THz radiation to provide a non-destructive assay of in situ leaf water content. When combined with commercially available psychrometers, it has the potential to offer a highly automated means of determining leaf tissue water relations parameters. The PV curves measured using the THz/psychrometer system show a high level of consistency because a single leaf is tracked continuously while avoiding the requirement for excessive handling of the sample, and particularly repeated cycles of pressurisation and depressurisation (as is encountered when using the Scholander pressure chamber). This application of THz radiation in conjunction with psychrometry also opens up the possibility of conducting PV curves in situ, thus allowing for greater insights into the regulation of cellular water relations while leaves remain connected to the plant hydraulic pathway.

## References

[1] Grace, J. Plant Water Relations. in *Plant Ecology*. (Wiley, Hoboken, 2009).

[2] Kirkham MB (2014). Principles of Soil and plant water relations.

[3] Philip JR (1966). Plant water relations: some physical aspects. Ann. Rev. Plant Physiol..

[4] Slavik B (1974). Methods of Studying Plant Water Relations.

[5] Bartlett MK, Scoffoni C, Sack L (2012). The determinants of leaf turgor loss point and prediction of drought tolerance of species and biomes: a global meta-analysis. Ecol. Lett..

[6] Gardner WR, Ehlig CF (1965). Physical aspects of the internal water relations of plant leaves. Plant Physiol..

[7] Tyree MT, Hammel HT (1972). The measurement of the turgor pressure and the water relations of plants by the pressure-bomb technique the measurement of the turgor pressure and the water relations of plants by the pressure-bomb technique. J. Exp. Bot..

[8] Smart RE, Bingham GE (1974). Rapid estimates of relative water content. Plant Physiol..

[9] Scholander PF, Bradstreet ED, Hemmingsen EA, Hammel HT (1965). Sap pressure in vascular plants. Science.

[10] Girona J, Mata M, Del Campo J, Arbonés A, Bartra E, Marsal J (2006). The use of midday leaf water potential for scheduling deficit irrigation in vineyards. Irrig. Sci..

[11] Hewitt F, Hough T, O'Neill P, Sasse J, Williams E, Rowan K (1985). Who taught plants thermodynamics? The unfulfilled potential of plant water potential. Austral. J. Plant Physiol..

[12] Kikuta SB, Kyriakopoulous E, Richter H (1985). Leaf hygrometer v. pressure chamber: a comparison of pressure–volume curve data obtained on single leaves by alternating measurements. Plant Cell Environ..

[13] Boyer J (1995). Measuring the Water Status of Plants and Solids.

[14] Puritch GS, Turner JA (1973). Effects of pressure increase and release on temperature within a pressure chamber used to estimate plant water potential. J. Exp. Bot..

[15] Coffey WLP, Gordon RJ, Dixon M (1997). Patterns of stem water potential in field grown potatoes using stem psychrometers. Potato Res..

[16] Turner NC, Spurway RA, Schulze ED (1984). Comparison of water potentials measured by in situ psychrometry and pressure chamber in morphologically different species. Plant Physiol..

[17] Martinez EM, Cancela JJ, Cuesta TS, Neira XX (2011). Review. Use of psychrometers in field measurements of plant material: accuracy and handling difficulties. Span. J. Agric. Res..

[18] Murphy R, Smith JAC (1994). A critical comparison of the pressure-probe and pressure-chamber techniques for estimating leaf-cell turgor pressure in Kalanchoe-Daigremontiana. Plant Cell Environ..

[19] Búrquez A (1987). Leaf thickness and water deficit in plants: a tool for field studies. J. Exp. Bot..

[20] McBurney T (1992). The relationship between leaf thickness and plant water potencial. J. Exp. Bot..

[21] Bartlett MK (2012). Rapid determination of comparative drought tolerance traits: Using an osmometer to predict turgor loss point. Methods Ecol. Evol..

[22] Wullschleger SD, Dlxonf MA (1988). Field measurement of leaf water potential with a temperature-corrected in situ thermocouple psychrometer. Plant Cell Environ.

[23] Dixon MA, Tyree MT (1984). A new stem hygrometer, corrected for temperature gradients and calibrated against the pressure bomb. Plant Cell Environ..

[24] Baldacci L (2017). Non-invasive absolute measurement of leaf water content using terahertz quantum cascade lasers. Plant Methods.

[25] Breitenstein B (2012). Introducing terahertz technology into plant biology: a novel method to monitor changes in leaf water status. J. Appl. Bot. Food Qual..

[26] Castro-Camus E, Palomar M, Covarrubias AA (2013). Leaf water dynamics of Arabidopsis thaliana monitored in-vivo using terahertz time-domain spectroscopy. Sci. Rep..

[27] Gente R (2013). Determination of leaf water content from terahertz time-domain spectroscopic data. J. Infrared Millimeter Terahertz Waves..

[28] Gente R, Koch M (2015). Monitoring leaf water content with THz and sub-THz waves. Plant Methods.

[29] Nie P (2017). Detection of water content in rapeseed leaves using terahertz spectroscopy. Sensors.

[30] Santesteban LG, Palacios I, Miranda C, Iriarte JC, Royo JB, Gonzalo R (2015). Terahertz time domain spectroscopy allows contactless monitoring of grapevine water status. Front. Plant Sci..

[31] Browne M, Yardimci NT, Scoffoni C, Jarrahi M, Sack L (2020). Prediction of leaf water potential and relative water content using terahertz radiation spectroscopy. Plant Direct..

[32] Xu J, Plaxco KW, Allen SJ (2006). Absorption spectra of liquid water and aqueous buffers between 0.3 and 3.72 THz. J. Chem. Phys..

[33] Edwards T, Walsh D, Spurr M, Rae C, Dunn M, Browne P (2006). Compact source of continuously and widely-tunable terahertz radiation. Opt. Express.

[34] Lee A, He Y, Pask H (2013). Frequency-tunable THz source based on stimulated polariton scattering in Mg:LiNbO3. IEEE J. Quantum Electron..

[35] Ortega TA, Pask HM, Spence DJ, Lee AJ (2018). Tunable 3–6 THz polariton laser exceeding 0.1 mW average output power based on crystalline RbTiOPO4. IEEE J. Sel. Top. Quantum Electron..

[36] Stothard DJM (2008). Line-narrowed, compact, and coherent source of widely tunable terahertz radiation. Appl. Phys. Lett..

[37] Lee A, Spence D, Pask H (2017). Tunable THz polariton laser based on 1342 nm wavelength for enhanced terahertz wave extraction. Opt. Lett..

[38] Jordens C, Scheller M, Breitenstein B, Selmar D, Koch M (2009). Evaluation of leaf water status by means of permittivity at terahertz frequencies. J. Biol. Phys..

[39] Kocsis L, Herman P, Eke A (2006). The modified Beer–Lambert law revisited. Phys. Med. Biol..

[40] Lucas WJ (2013). The plant vascular system: evolution, development and functions. J. Integr. Plant Biol..

[41] Datt B (1999). Remote sensing of water content in eucalyptus leaves. Aust. J. Bot..

[42] Koide RT, Robichaux RH, Morse SR, Smith CM, Pearcy RW, Ehleringer J, Mooney HA, Rundel PW (2000). Plant Water Status, Hydraulic Resistance and Capacitance. Plant Physiological Ecology: Field Instrumentation and Methods.

[43] Richter H (1978). A diagram for the description of water relations in plant cells and organs. J. Exp. Bot..

[44] Patakas A, Nikolaou N, Zioziou E, Radoglou K, Noitsakis B (2002). The role of organic solute and ion accumulation in osmotic adjustment in drought-stressed grape vines. Plant Sci..

[45] Rodrigues ML (1993). Osmotic adjustment in water stressed grapevine leaves in relation to carbon assimilation. Funct. Plant Biol..

[46] Bartholomeus RP, Witte JPM, Van Bodegom PM, Van Dam JC, Aerts R (2011). Climate change threatens endangered plant species by stronger and interacting water-related stresses. J. Geophys. Res.: Biogeosci..

[47] Franks PJ, Buckley TN, Shope JC, Mott KA (2001). Guard cell volume and pressure measured concurrently by confocal microscopy and the cell pressure probe. Plant Physiol..

[48] Tomos AD, Steudle E, Zimmermann U, Schulze ED (1981). Water relations of leaf epidermal-cells of Tradescantia-Virginiana. Plant Physiol..

